# Supplementary figures and images for: Impaired host resistance to Salmonella during helminth co-infection is restored by anthelmintic treatment prior to bacterial challenge
Source: PLoS Negl Trop Dis. 2021 Jan 20;15(1):e0009052. doi: 10.1371/journal.pntd.0009052 (PMC7850471; doi:10.1371/journal.pntd.0009052)

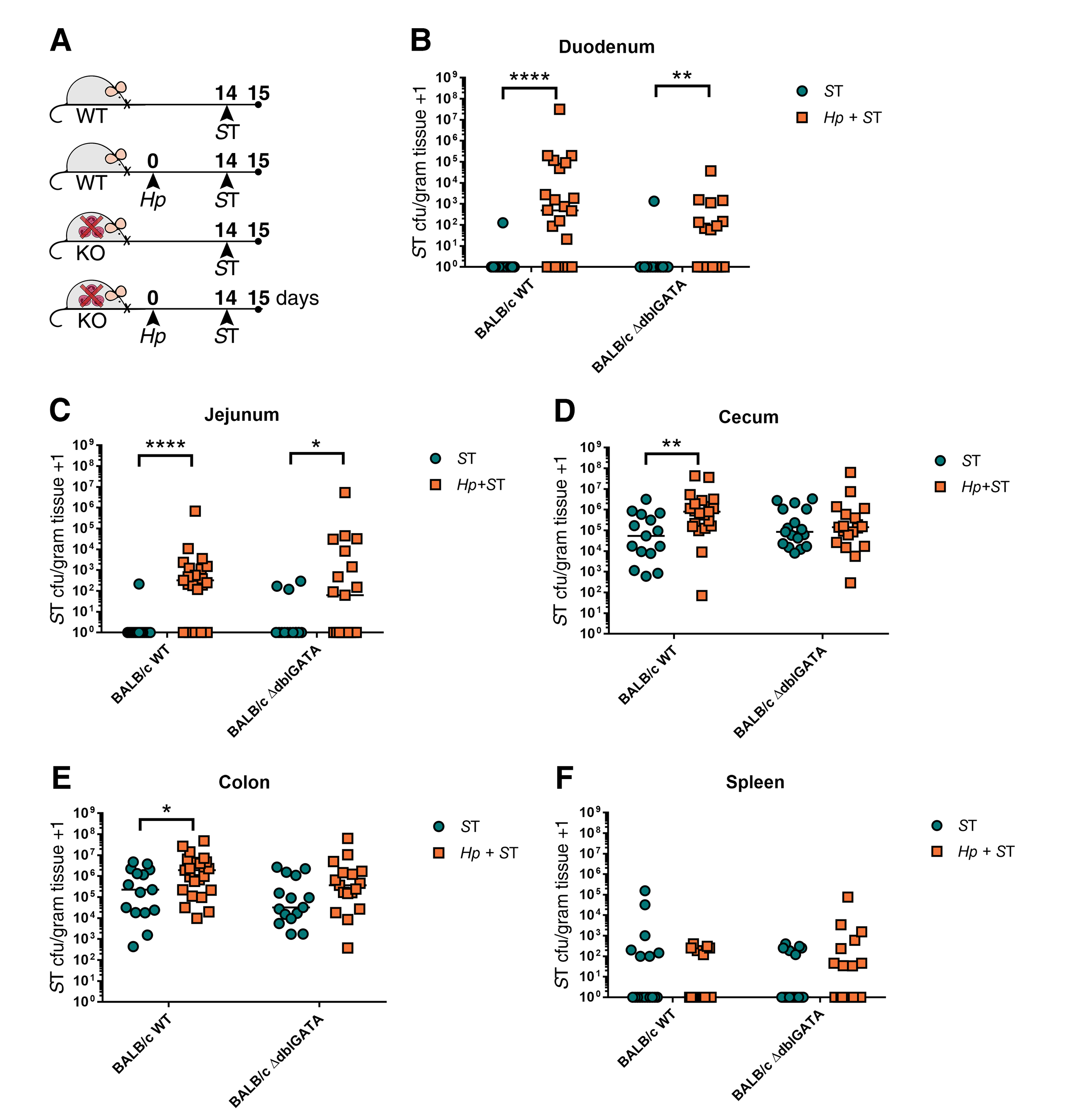

Supplement: S1 Fig — (A) Experimental set-up. Naïve or H. polygyrus (Hp)-infected wild-type and eosinophil-deficient ΔdblGATA BALB/cJ mice were orally infected with ΔaroA ST fourteen days post Hp-infection. One day post-ST infection, ST colony-forming units (cfu)/gram of tissue were determined. ST cfu/gram of tissue in the duodenum (B), jejunum (C), cecum (D), colon (E), and spleen (F) are shown. Data shown are pooled from three independent experiments including both male and female mice. Statistical comparisons for each mouse genotype were made using a Mann-Whitney test. A line indicates the median value for each experimental group. * = p ≤ 0.05 ** = p ≤ 0.01; **** = p ≤ 0.0001. (TIF) [file pntd.0009052.s002.tif]

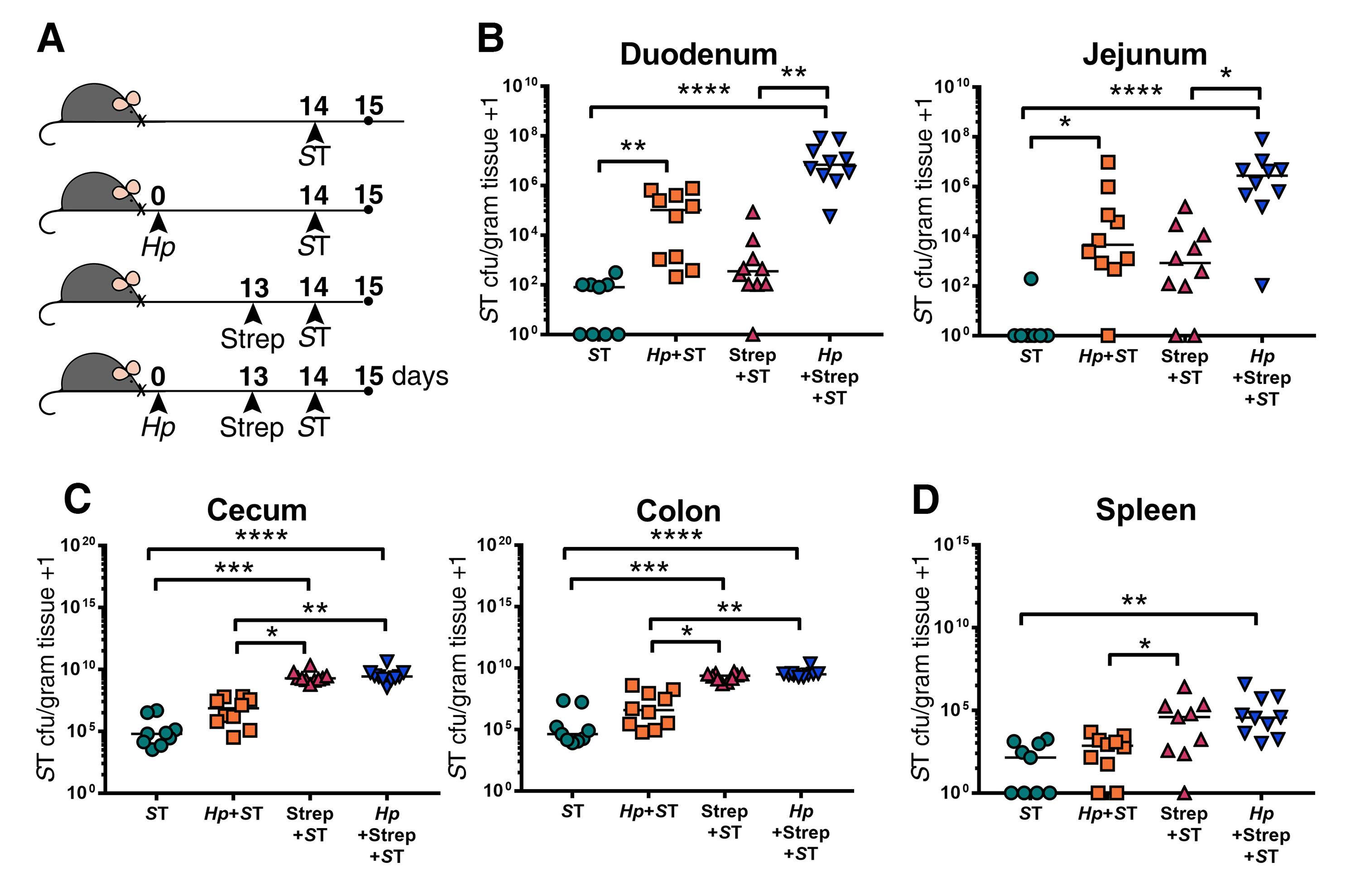

Supplement: S2 Fig — (A) Experimental set-up. Naïve or H. polygyrus (Hp)-infected male and female C57BL/6J mice were given a 20 mg dose of streptomycin by oral gavage thirteen days post Hp-infection, or left untreated. One day following treatment, all mice were infected with ΔaroA ST. One day post-ST infection, ST colony-forming units (cfu)/gram of tissue were determined. ST cfu/gram of tissue in the duodenum and jejunum (B), cecum and colon (C), and spleen (D) are shown. Data shown are pooled from two independent experiments. Statistical comparisons between groups were made using a Kruskal-Wallis test followed by a Dunn’s multiple comparisons test. A line indicates the median value for each experimental group. * = p ≤ 0.05; ** = p ≤ 0.01; *** = p ≤ 0.001; **** = p ≤ 0.0001. (TIF) [file pntd.0009052.s003.tif]

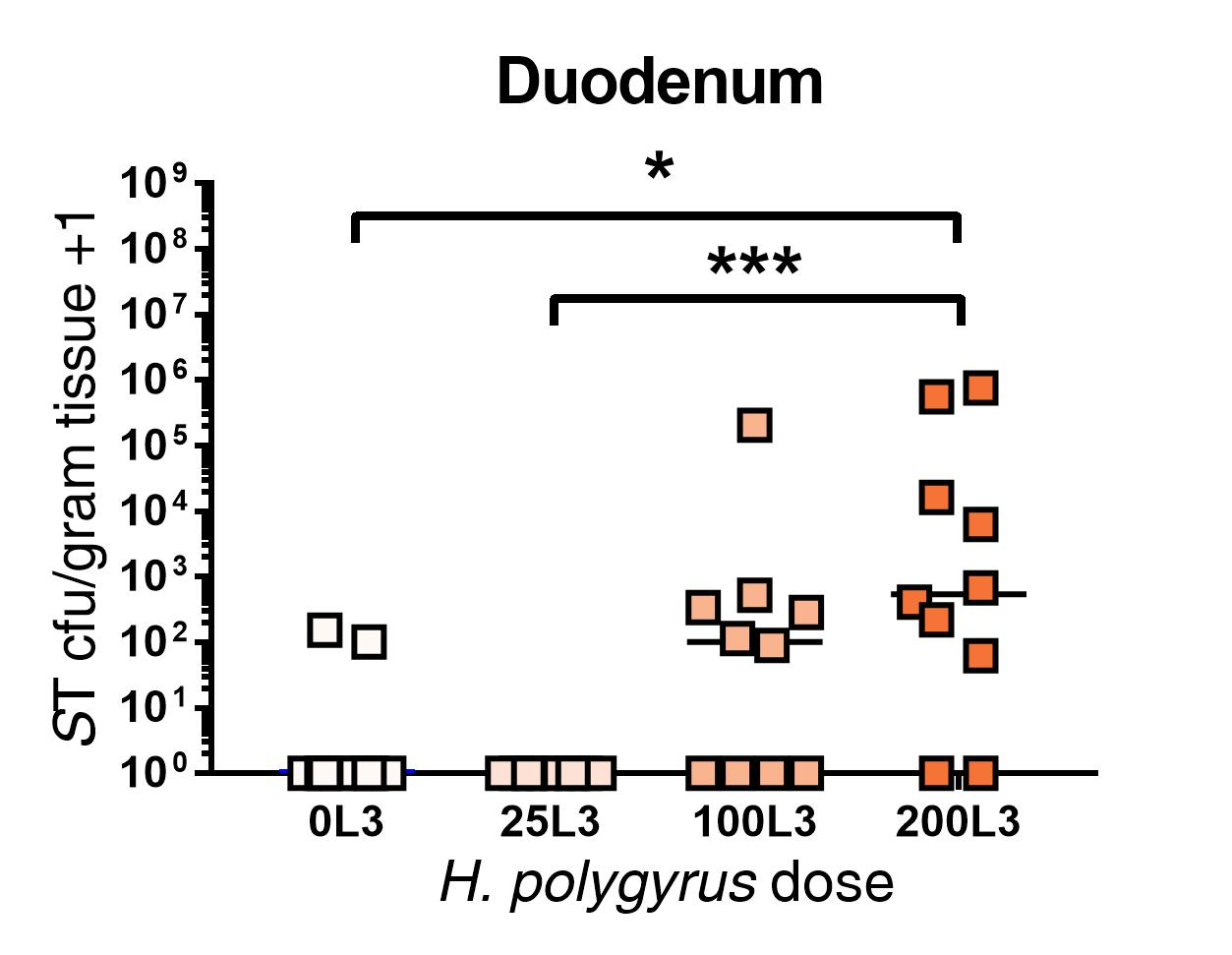

Supplement: S3 Fig — Female C57BL/6J mice were left naïve (‘0L3’) or infected with 25 (‘25L3’), 100 (‘100L3’), or 200 (‘200L3’) third stage H. polygyrus larvae (L3). Fourteen days later, mice were orally infected with ΔaroA ST. One day post-ST infection, ST colony-forming units (cfu)/gram of tissue were determined in the duodenum. Data shown are pooled from two independent experiments. Statistical comparisons between groups were made using a Kruskal-Wallis test followed by a Dunn’s multiple comparisons test. A line indicates the median value for each experimental group. * = p ≤ 0.05; *** = p ≤ 0.001. (TIF) [file pntd.0009052.s004.tif]

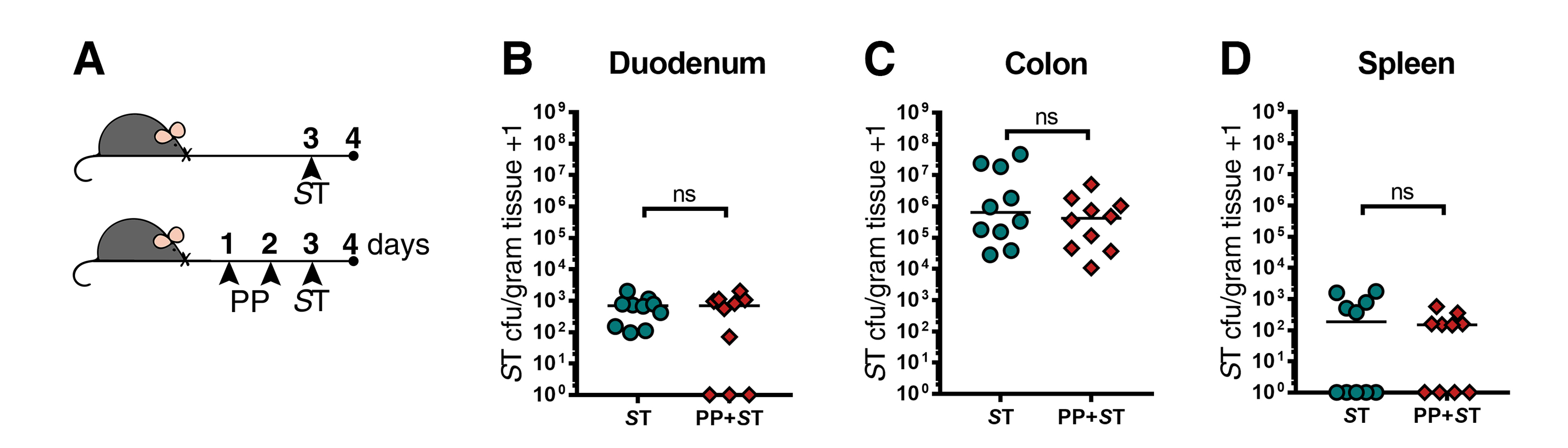

Supplement: S4 Fig — (A) Experimental set-up. Naïve male and female C57BL/6J mice were treated with 2.5 mg Strongid P for two consecutive days or left untreated. One day after completion of PP treatment, mice were infected with wild-type ST to assess ST colony forming units (cfu) in the duodenum (B), or mice were infected with ΔaroA ST to assess ST cfu in the colon (C) and spleen (D). We used wild-type ST to assess the effect of PP on ST in the duodenum, rather than ΔaroA ST, because ΔaroA ST establishes at only low levels in the small intestine in the absence of helminths, making it impossible to detect a potentially adverse effect of PP on ST colonization in the small intestines. Wild-type ST is able to establish sufficiently in the small intestine, which allows us to determine whether PP affects colonization levels of ST. Data shown in both (B) and (C+D) are pooled from two independent experiments, and statistical comparisons between groups were made using a Mann-Whitney test. A line indicates the median value for each experimental group. ns = not significant. (TIF) [file pntd.0009052.s005.tif]

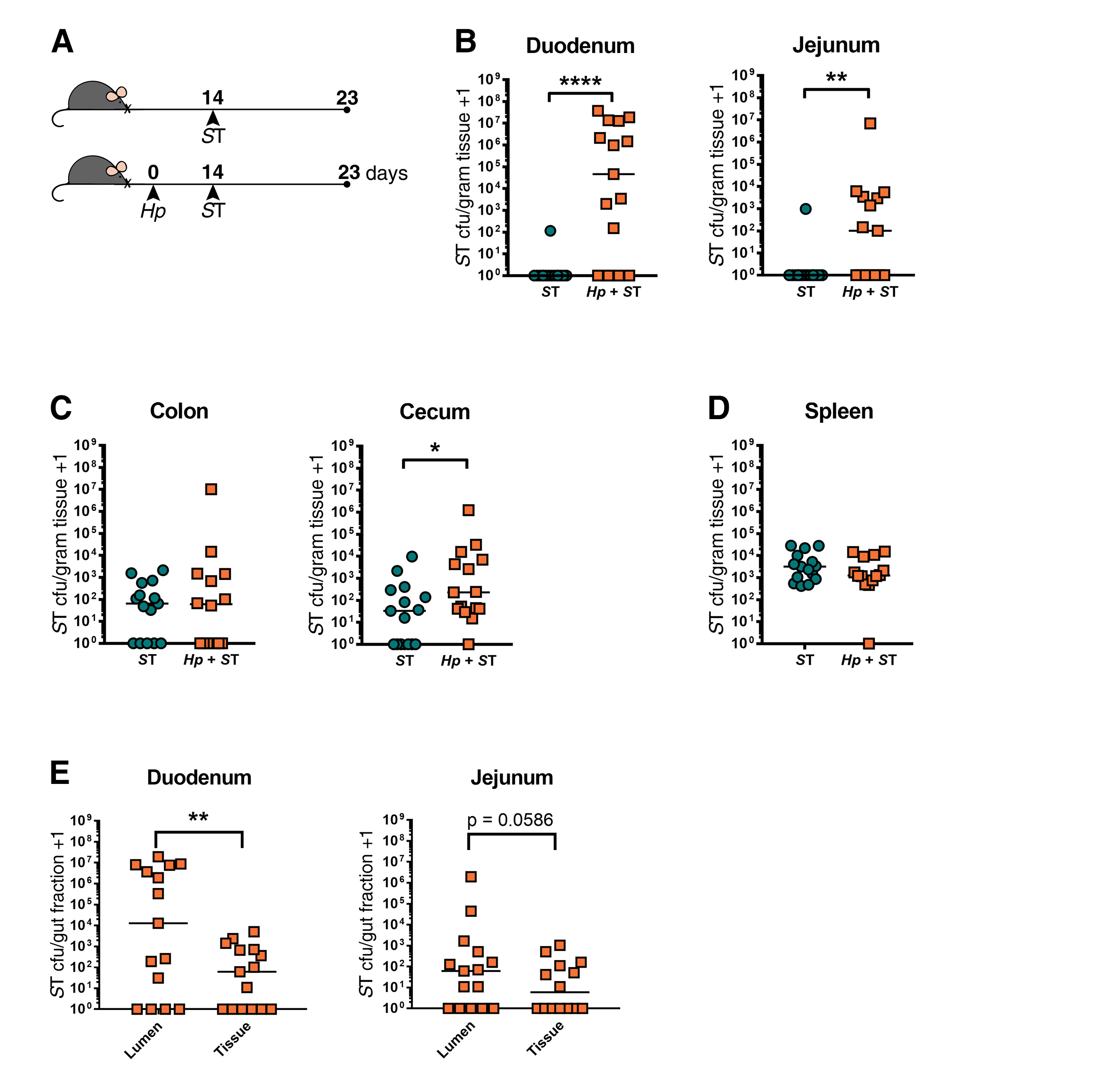

Supplement: S5 Fig — (A) Experimental set-up. Naïve or H. polygyrus (Hp)-infected male and female C57BL/6J mice were orally infected with ΔaroA ST fourteen days post Hp-infection. Nine days post-ST infection, ST colony-forming units (cfu)/gram of tissue were determined. ST cfu/gram of tissue in the duodenum and jejunum (B), cecum and colon (C), and the spleen (D) are shown. Data shown are pooled from three independent experiments. Statistical comparisons between groups were made using a Mann-Whitney test. In a different set of experiments following the same experimental timeline, the duodenum and jejunum were dissected to separate out tissue and luminal fractions, and ST colony-forming units (cfu) were determined in each fraction (E). Data shown are pooled from three independent experiments. Statistical comparisons between groups were made using a Wilcoxon matched-pairs signed rank test. A line indicates the median value for each experimental group. * = p ≤ 0.05 ** = p ≤ 0.01; **** = p ≤ 0.0001. (TIF) [file pntd.0009052.s006.tif]

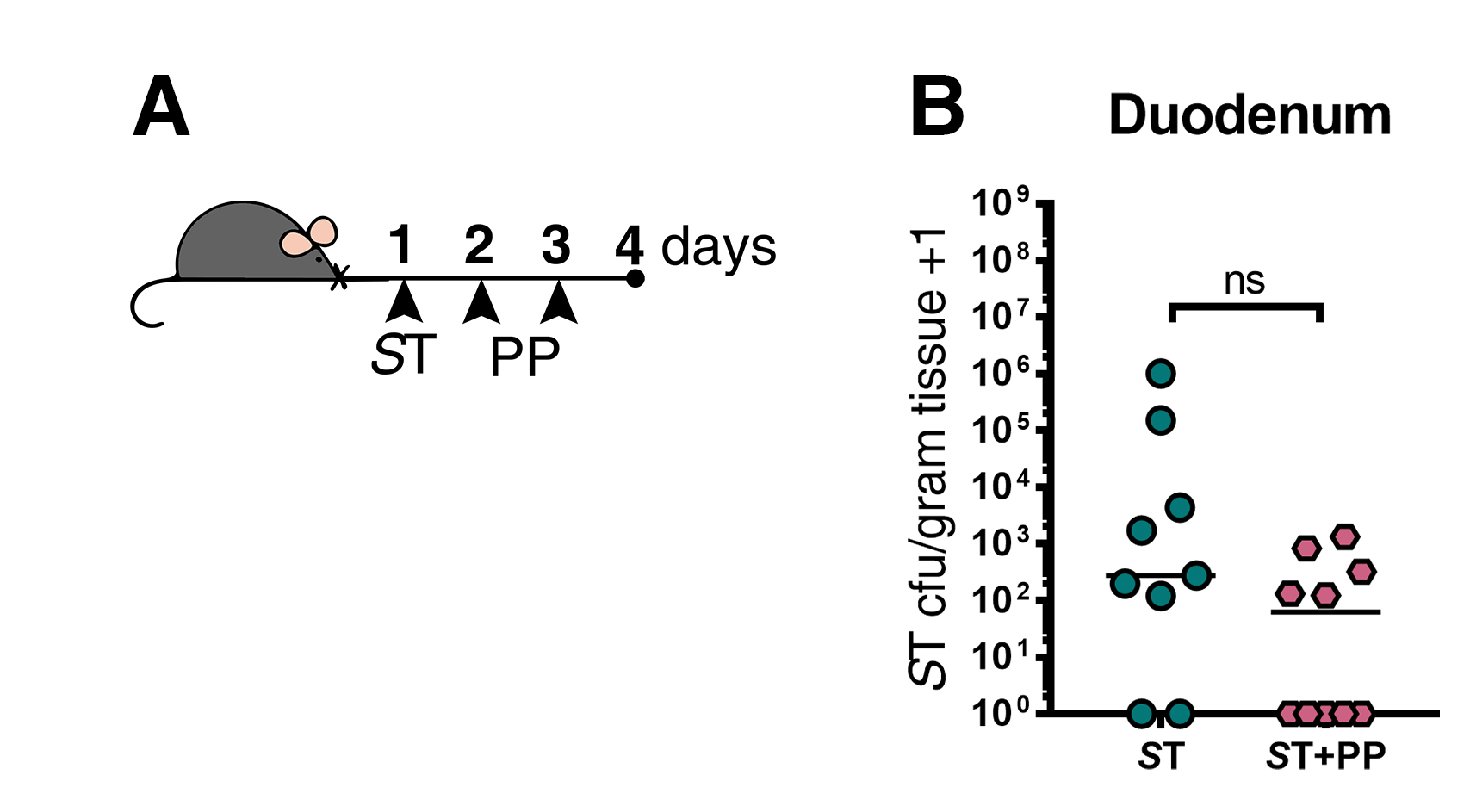

Supplement: S6 Fig — Male C57BL/6J mice were infected with wild-type ST. On the first and second day after ST infection, mice were treated with a 2.5 mg dose of Strongid P or left untreated. One day after completion of PP treatment, ST colony-forming unit (cfu) counts were determined in the duodenum. The purpose of this experiment was to test whether PP treatment had any effect on ST colonization levels in the small intestine. Because we were looking to detect a potential reduction in ST burdens following PP treatment, we used wild-type ST rather than ΔaroA ST, since wild-type ST colonizes to higher levels in the small intestine which would allow us to detect a potential reduction in colonization after PP treatment. Data shown are pooled from two independent experiments. Statistical comparisons between groups were made using a Mann-Whitney test. A line indicates the median value for each experimental group. ns = not significant. (TIF) [file pntd.0009052.s007.tif]

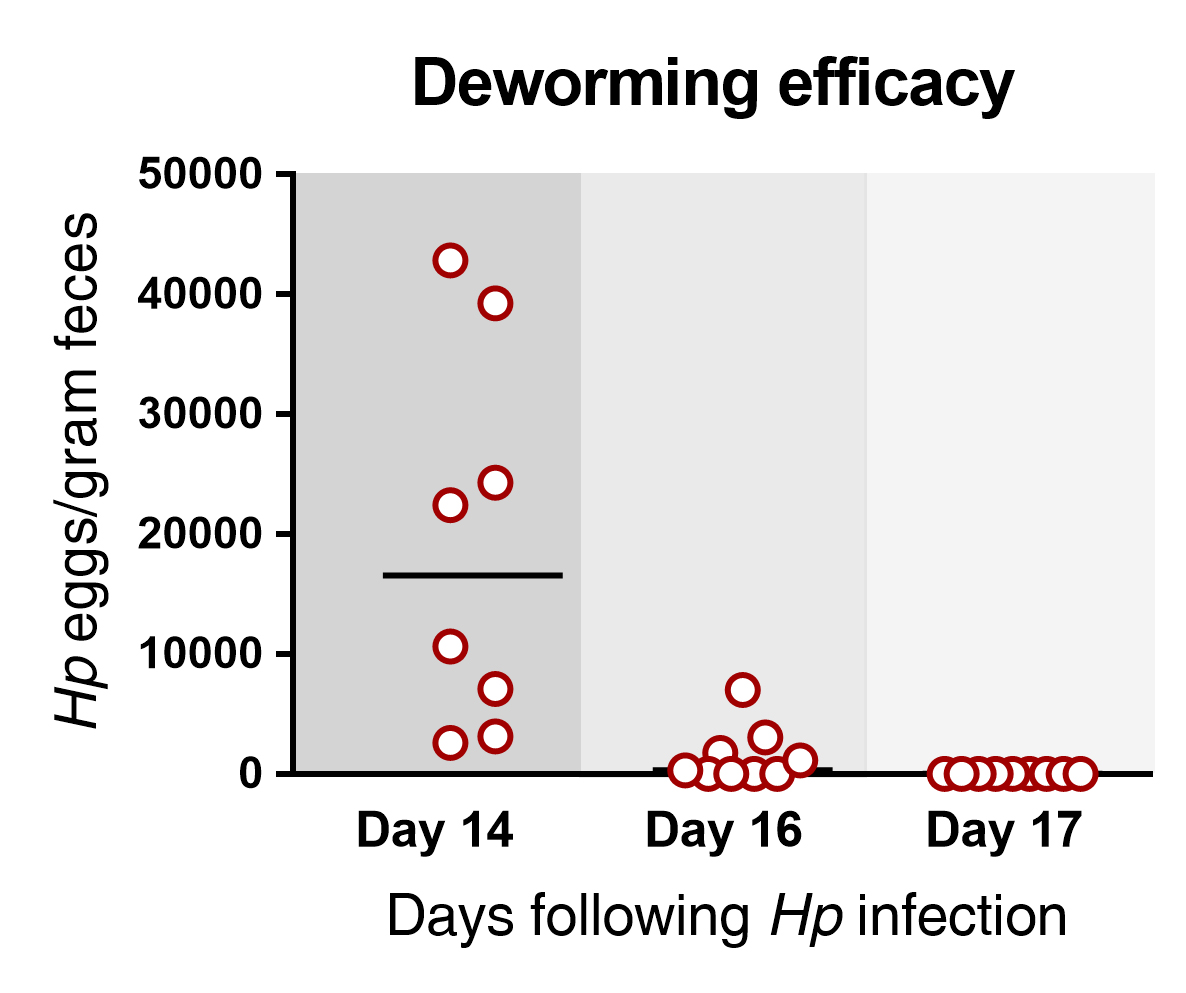

Supplement: S7 Fig — Numbers of Hp eggs released in feces were quantified on days 14–16 from mice receiving deworming treatment, as a non-terminal method of assessing worm burdens, to confirm anthelmintic treatment efficacy. (TIF) [file pntd.0009052.s008.tif]

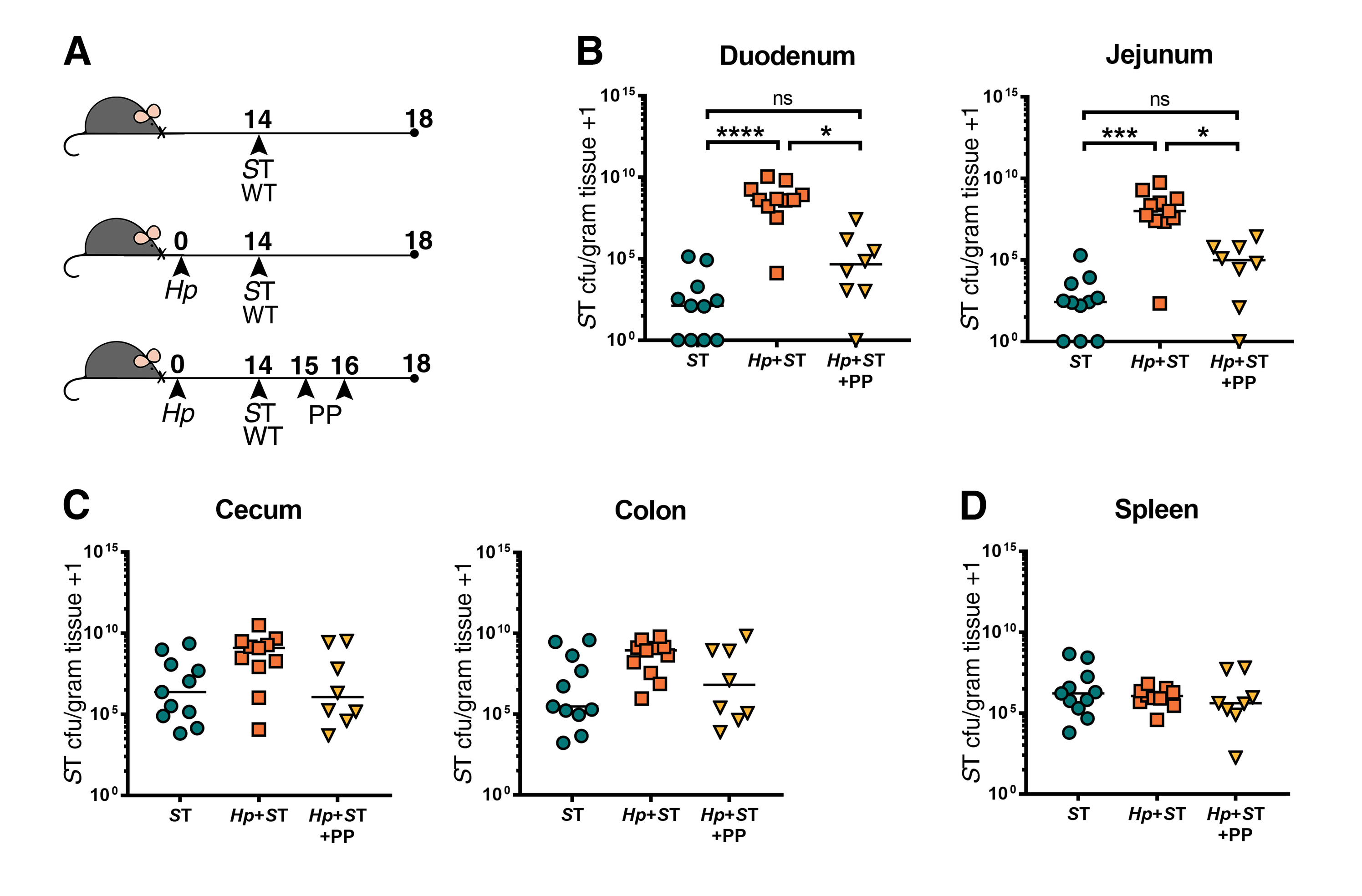

Supplement: S8 Fig — (A) Experimental set-up. Naïve or H. polygyrus (Hp)-infected female C57BL/6J mice were orally infected with wild-type ST fourteen days post-Hp infection. One day post-ST infection, Hp-co-infected mice were given deworming treatment (PP) for two days or not. Two days post-treatment or no treatment, ST colony-forming units (cfu)/gram of tissue were determined in all groups. ST cfu/gram of tissue in the duodenum and jejunum (B), cecum and colon (C), and the spleen (D) are shown. Data shown are pooled from two independent experiments. Statistical comparisons between groups were made using a Kruskal-Wallis test followed by a Dunn’s multiple comparisons test. A line indicates the median value for each experimental group. ns = not significant; * = p ≤ 0.05; *** = p ≤ 0.001; **** = p ≤ 0.0001. (TIF) [file pntd.0009052.s009.tif]

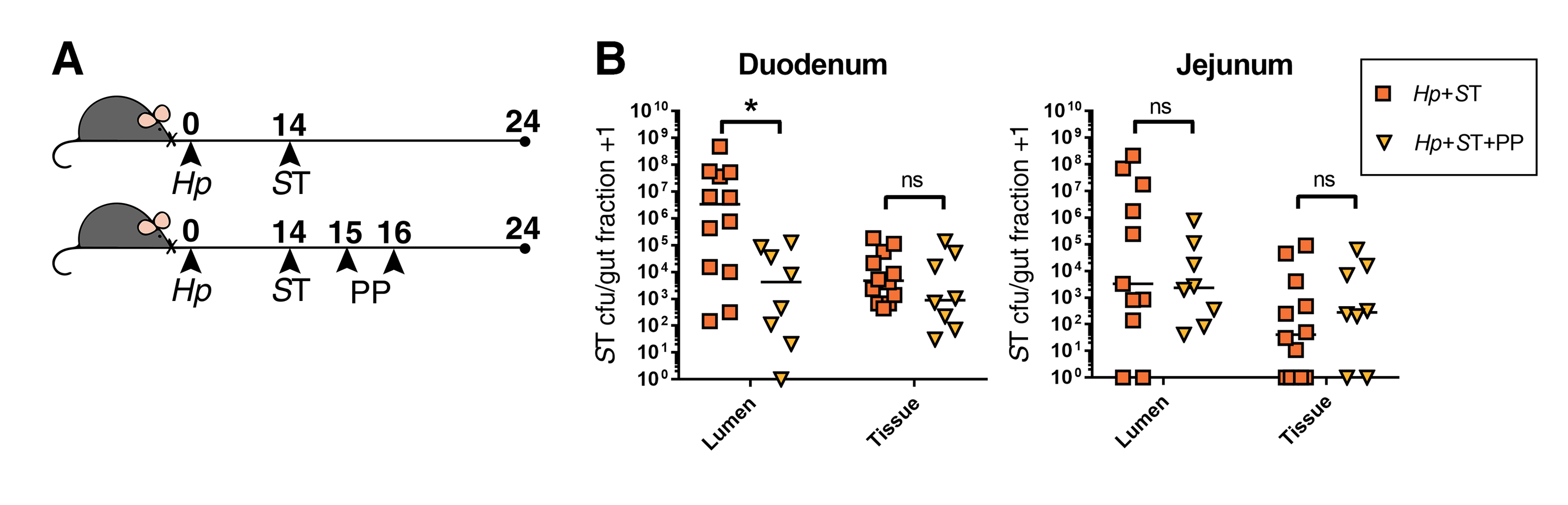

Supplement: S9 Fig — (A) Experimental set-up. Male and female C57BL6/J mice were infected with H. polygyrus (Hp). Fourteen days post-Hp infection, mice were orally infected with ΔaroA ST. One day post-ST infection, Hp-co-infected mice were given deworming treatment (PP) for two days or not. Eight days post-treatment or no treatment, ST colony-forming units (cfu)/gram of tissue were determined. (B) ST cfu in luminal and tissue small intestinal fractions. Data shown are pooled from two independent experiments. Statistical comparisons were made between the indicated groups using a Mann-Whitney test. A line indicates the median value for each experimental group. * = p ≤ 0.05. (TIF) [file pntd.0009052.s010.tif]
