# Supplementary material for: Impaired host resistance to Salmonella during helminth co-infection is restored by anthelmintic treatment prior to bacterial challenge
Source: PLoS Negl Trop Dis. 2021 Jan 20;15(1):e0009052. doi: 10.1371/journal.pntd.0009052 (PMC7850471; doi:10.1371/journal.pntd.0009052)
Supplement: Attachment — Submitted filename: Response to reviewers.pdf [file pntd.0009052.s011.pdf]

University of Victoria
Dr. Lisa A. Reynolds | Dept. Biochemistry & Microbiology
PO Box 1700 STN CSC, Victoria, BC, V8W 2Y2, Canada
T 250-721-6658 | lisareynolds@uvic.ca| https://oac.uvic.ca/reynoldslab

October 31, 2020

Manuscript PNTD-D-20-00912

Dear Editors,

Thank you for considering our manuscript '**Impaired host resistance to Salmonella during helminth co-infection is restored by anthelmintic treatment prior to bacterial challenge**' by Tara Brosschot *et al*. We were pleased to receive your email on Aug 24, 2020, containing Reviewer's comments and inviting us to resubmit a revised version of our manuscript.

We would like to thank the Reviewer's for providing us with thoughtful and constructive feedback. We have responded to each point raised, and consequently we have extended our manuscript (**S1A-F, S2A-D, S7, S8A-D, S9A-B are all newly added data**), and amended the text of our manuscript to include these new data and make clarifications/adjustments as per the Reviewer's suggestions. We have marked all amendments to our manuscript with yellow highlighting, and all the changes we have made in response to the Reviewer's comments are fully detailed below.

As instructed in your email on Aug 24, 2020, we have uploaded all of our figure files to the PACE tool and confirmed that they meet journal specifications. We have also included a file with our resubmission containing all data used to generate each of our figures, which is included as a supporting information excel file '**Data S1**'.

Reviewer's Responses to Questions
As you describe the new analyses required for acceptance, please consider the following:

Methods
-Are the objectives of the study clearly articulated with a clear testable hypothesis stated?
-Is the study design appropriate to address the stated objectives?
-Is the population clearly described and appropriate for the hypothesis being tested?
-Is the sample size sufficient to ensure adequate power to address the hypothesis being tested?
-Were correct statistical analysis used to support conclusions?
-Are there concerns about ethical or regulatory requirements being met?

Reviewer #1: yes
Reviewer #2: The authors provide a rigorous and clear study design with appropriate controls in the main and supplemental figures, and sample size was appropriate.

Many thanks for your positive assessment of our methodology.

Results
-Does the analysis presented match the analysis plan?
-Are the results clearly and completely presented?
-Are the figures (Tables, Images) of sufficient quality for clarity?

Reviewer #1: yes

Many thanks for your positive assessment.

Reviewer #2: For the most part: Results are clearly presented and easy to follow. Quality of the graphs are high.
Some validation data that are lacking in the deworming figures are fecal egg burdens of untreated vs PP-treated mice:
- In fig 1, it seems the data is not shown for day 15 and 16 of Hp alone (unless those mice cleared Hp?). This is a necessary control.

We have reviewed the way that we presented our data to validate the efficacy of deworming treatment and agree that the way we presented this data initially was confusing. In the panel where we presented *H. polygyrus* egg data in our originally submitted version of Figure 1A, no data points were shown for day 15 and 16 of *H. polygyrus* alone, as the reviewer correctly notes. Our intention was for this figure to convey two pieces of information:
1) Most importantly, that our drug clearance was effective, as evidenced by a lack of *H. polygyrus* eggs in the feces of the *H. polygyrus* + anthelmintic treatment ['*Hp* + PP'] after two days of drug clearance, which began on day 14 following *H. polygyrus* infection.
2) That our two experimental groups, *H. polygyrus*-infected with no anthelmintic treatment [Hp] and *H. polygyrus* infected with subsequent anthelmintic treatment ['*Hp* + PP'], *initially* had equivalent infectious doses of *H. polygyrus* on day 14 of *H. polygyrus* infection, which is the timepoint when anthelmintic drugs began to be administered to the *Hp* + PP group.
We did not show *H. polygyrus* egg data for the '*Hp*' group at day 15 or 16 of *H. polygyrus* infection since this group was infected with *Salmonella* on day 14 of *H. polygyrus* infection and euthanized on day 15 following *H. polygyrus* infection.
We have now revised the panel in Figure 1A where we present egg burden data, to only show data from the '*Hp* + PP' group, since our main intention was to provide evidence of the efficacy of anthelmintic treatment. To ensure our readers know that we confirmed that the infectious dose of *H. polygyrus* was equivalent between the '*Hp*' and *'Hp* + PP' groups prior to beginning anthelmintic treatment, we make a statement to this effect in the Methods section of our manuscript on **page 7 lines 101-104**.

- In Fig 4, egg burden data for Hp and Hp+PP is needed.

We now include data evidencing the efficacy of our anthelmintic treatment regime for experiments presented in Figure 4 in **new Figure S7**. For similar reasons to those outlined in our

point above, we have not included egg data from the '*Hp*' group in this figure to avoid confusion as to why egg count data is not shown at days 15 and 16 following *H. polygyrus* infection from this group. The main need for including egg burden data for these experiments are to confirm the efficacy of anthelmintic treatment in the '*Hp* + PP' group, and our newly added statement on **page 7 lines 101-104** confirms for readers that egg counts were equivalent between '*Hp*' and '*Hp* + PP' groups prior to beginning anthelmintic treatment.

Conclusions
-Are the conclusions supported by the data presented?
-Are the limitations of analysis clearly described?
-Do the authors discuss how these data can be helpful to advance our understanding of the topic under study?
-Is public health relevance addressed?

Reviewer #1: yes

Many thanks for your positive assessment.

Reviewer #2: The conclusion, that H.polygyrus impaired resistance to Salmonella requires ongoing H.polygyrus infection during bacterial inoculation, is supported by the experiments administering deworming PP before or after inoculation. These findings are of public health relevance for co-infections and understanding the consequences of pre-existing helminth infections for subsequent bacterial infections.

Many thanks for your positive assessment.

The authors address another point about the necessity for invasive Salmonella for this phenotype, however, these data are not complete to allow conclusions to be drawn. Required experiments would be to test invA mutant Salmonella in the experimental design for PP-deworming after bacterial inoculation.

Many thanks for this suggestion. We have now completed an additional set of experiments as suggested to test whether invA is required to allow *Salmonella* to persist in the murine small intestine after PP-deworming. The *invA* mutant we use throughout our manuscript has a wild-type (WT) background and this limits the maximum length of experiments which are possible with this strain because mice start to succumb to WT *Salmonella* infection typically ~4 days after inoculation. With this in mind, we designed our new experiments with an endpoint at 4 days post *Salmonella* infection. Our new experiments included mice infected with WT *Salmonella* in comparison to mice infected with *invA* mutant *Salmonella.* We co-infected mice with helminths and *Salmonella*, then administered PP deworming treatment, and then determined *Salmonella* burdens 4 days following *Salmonella* infection. Our controls were mice singly infected with *Salmonella* and mice co-infected with helminths and *Salmonella* that did not receive PP deworming. See experimental timeline:

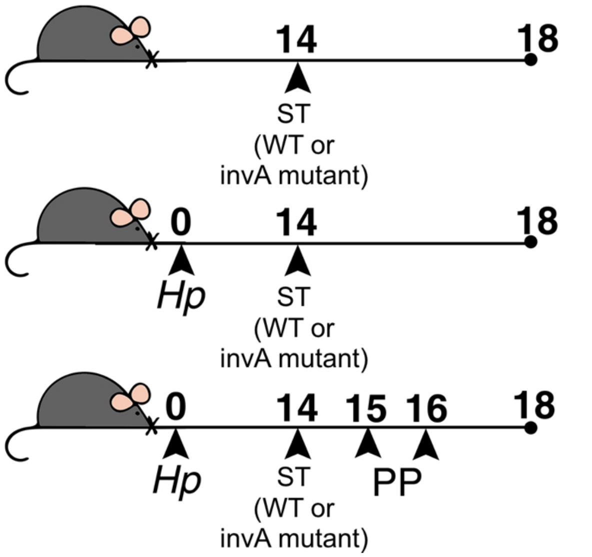

Figure A

We were expecting to be able to answer the question: is invA required for *Salmonella* to persist in the small intestine after helminths have been cleared? (In other words- does *Salmonella* need to be able to invade host tissue in order to persist in the small intestine after helminths have been drug cleared?)
This experimental setup did not allow us to obtain a clear answer to this question. We present the results combined from two experimental repeats of this experiment below. *Salmonella* cfu counts in the duodenum:

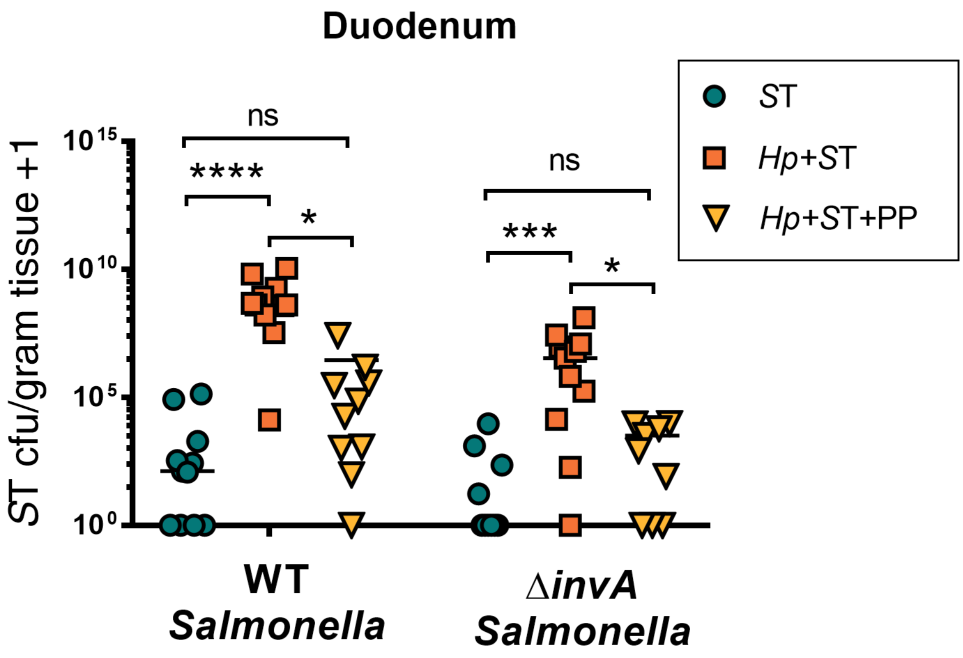

Figure B

As you can see, for both WT and *invA* mutant *Salmonella, Salmonella* burdens are elevated in helminth co-infected mice, compared to mice singly infected with *Salmonella.* Administration of

anthelmintics (PP) significantly reduces *Salmonella* burdens, compared to helminth infected mice that did not receive PP, regardless of the *Salmonella* strain used for infections. However, at this timepoint, and with a wildtype *Salmonella* background, we found no significant difference in *Salmonella* burdens between mice singly infected with *Salmonella* (*S*T) and helminth co-infected and anthelmintic treated (*Hp*+*S*T+PP) mice. Since we saw no significant differences between these groups, we do not have evidence from this experiment to suggest that following helminth clearance, WT *Salmonella* persists in the small intestine to higher levels than if helminths were never present. Thus, asking whether invA is required to support this persistence in the small intestine becomes redundant.
In our manuscript Figures 3 and 4 we use the slower growing, aroA strain of *Salmonella*, which allows us to explore extended timepoints following *Salmonella* infection. These data similarly show that only at later timepoints is there evidence of a population of *Salmonella* which 'persists' in the small intestine following helminth clearance (there are significantly higher levels of *Salmonella* in the small intestine of mice that had been helminth co-infected and subsequently anthelminthic treated than in mice singly infected with *Salmonella*, Figure 4B).

Given the fact that in our newly conducted experiments (*Figure B*, above) the invA comparison is redundant, we decided not to include the invA data in our revised manuscript, and have included it above only for your reference. However, we have incorporated the data we obtained from using the WT strain of *Salmonella* into our manuscript as **new Figure S8**, which complement and extend upon existing data in our manuscript shown in Figure 3+4. We reference this new data on **page 14, line 260 – page 15 line 265** of our revised manuscript.

We also performed an alternative set of new experiments that would better address your comment as to whether tissue invasion by *Salmonella* was required for a population of *Salmonella* to persist in the small intestine after helminth clearance. These new data are now presented as **new Figure S9**. In these experiments, we used the aroA strain of *Salmonella* to allow us to explore later timepoints following *Salmonella* infection, as we did in Figure 4. These new data show that after anthelmintic treatment of helminth co-infected mice, the luminal fraction of *Salmonella* is reduced, rather than the fraction of *Salmonella* which has invaded host tissue.

The text of our manuscript has been adjusted between **page 14 line 260 and page 16 line 292** to incorporate these new data.

We would like to thank the reviewer for suggesting these additional experiments, which has led to clarification of the phenotype with PP-deworming after *Salmonella* infection and enabled us to more thoroughly define how anthelmintic treatment affects *Salmonella* infection dynamics.

Editorial and Data Presentation Modifications?
Use this section for editorial suggestions as well as relatively minor modifications of existing data that would enhance clarity. If the only modifications needed are minor and/or editorial, you may wish to recommend "Minor Revision" or "Accept".

Reviewer #1: (No Response)

Reviewer #2: - Data is presented clearly and in a well-organized manner that makes it easy to follow.

Many thanks for your positive assessment.

- The relevance of figure 3 is unclear, and should be added to another figure, or in supplemental. Alternatively, this distribution should be considered in the context of the deworming treatments, or the use of the invA mutant.

We have now adjusted our Figures to present our tissue vs lumen data that was previously submitted in Figure 3 alongside data we obtained using the invA mutant. These data are now presented together in our revised Figure 2. We agree it makes sense to present these data together, since they are complementary: we find that during helminth infection *Salmonella* is predominately colonizing the small intestinal lumen, rather than invading host tissue, and our data from invA mutant *Salmonella* shows that as expected from this mutant which cannot invade host tissue, invA mutant *Salmonella* is still able to take advantage of a helminth-modified small intestinal environment to colonize the small intestinal lumen.

As discussed above, we have also now added new data in new **Figure S9** which reveals the distribution of *Salmonella* in the context of deworming treatment.

- I'd advise that Fig S5 be included

As suggested we have now added this Figure into the main manuscript Figures. This data is now presented as Figure 3.

Summary and General Comments
Use this section to provide overall comments, discuss strengths/weaknesses of the study, novelty, significance, general execution and scholarship. You may also include additional comments for the author, including concerns about dual publication, research ethics, or publication ethics. If requesting major revision, please articulate the new experiments that are needed.

Reviewer #1: The premise and theme of this work are important; how are helminths and bacteria interacting in the mammalian gut and what is the effect of anthelminthic treatment on colonization of the host with pathogenic bacteria.

We appreciate that the Reviewer is in agreement with us that it is important to understand how helminths and bacteria interact in the mammalian gut, and what the impact of anthelmintic treatment is on this relationship.

The manuscript is logical and clearly written. The data are clearly presented, with the appropriate statistical analyses and the conclusions are not over-stated. The discussion is long and could be cut by 25% (there is some redundancy in sentence structure).

As suggested we have now carefully edited our discussion section to avoid any redundancy and ensure that it is concise, reducing its overall length.

The authors have reproduced some of their previously published data (i.e. infection with H. polygyrus increases Salmonella colonization). Here they extend this by showing that: (1) clearance of H. polygyrus with an anthelminthic treatment prevents the enhanced colonization when Salmonella are inoculated; (2) the enhanced colonization by Salmonella is independent of the bacterium's invA gene; (3) if the anthelminthic is given after Salmonella infection, then enhanced colonization of the bacteria persists; and, (4) there appears to be a 'physical' interaction/association between the worm and the bacteria.
These are all robust observations. Therein lies the concern with this manuscript. The points the manuscript makes are well-taken, but the authors make no attempt at defining the mechanism underlying this helminth-bacteria interaction. They are clearly aware of this, because much of the discussion speculates on the mechanism(s) of this interaction. The data presented is a solid platform on which to build a mechanistic study. The manuscript in its current form does not go far enough – simple assays (worm/egg counts, bacterial culture/cfu enumeration) support some interesting observations. The impact of this research would be boosted by analyses of 'how' this happens, and even ruling possibilities out would be a valuable addition to the field.

Our manuscript is the first description of how anthelmintic treatment affects the influence of helminths on pathogenic bacterial colonization. The Reviewer summarizes our key findings accurately and we are pleased the Reviewer assesses our conclusions to be robust. We are aware that questions still remain as to the exact mechanism(s) underlying the helminth-bacteria interaction. The focus of our manuscript is on the impact of anthelmintic treatment on helminth-mediated enhancement of bacterial colonization, and providing a thorough description of the mechanisms driving helminth-mediated bacterial colonization is beyond the scope of our current study.
Our manuscript does provide novel mechanistic insights, notably that the helminth-mediated expansion of *Salmonella* occurs primarily in the small intestinal lumen, rather than through promoting proliferation of *Salmonella* within host cells, and *Salmonella* expression of invA is not required to take advantage of the helminth-modified intestinal environment. We think it is likely that multiple parallel mechanisms drive helminth-promotion of bacterial colonization, and multiple hypotheses would need to be tested in order to identify potential mechanisms.
This reviewer suggests that 'even ruling out possibilities would be a valuable addition to the field'. Our lab has and continues to test various hypotheses regarding the mechanism(s) that can contribute to enhanced colonization of the small intestine by pathogenic bacteria during helminth, and we have indeed 'ruled out' several possibilities. As a result of this Reviewer's suggestion we have now included some of these data in our current manuscript as supplemental data. We agree that ruling out possibilities is a valuable contribution to the field, but equally we do not want to distract too much from the current flow of our manuscript by presenting data which tests various disparate hypotheses, as we would like the emphasis of our manuscript to remain on our robust findings describing the impact of anthelmintic treatment

on bacterial persistence following helminth infection. We now include **new Figures S1 and S2** that reveal that the absence of eosinophils does not preclude the ability of *H. polygyrus* to promote *Salmonella* colonization, nor does depletion of the bacterial microbiota. We refer to this newly added data on **page 9 lines 147-150**, and also discuss this data in context with our other data in the discussion on **page 20 lines 376-386**. We also emphasize previously published mechanisms we have 'ruled out' in this newly edited section of our manuscript.

Finally, the authors note the host-parasite specificity of helminth-bacteria interactions and so it would be useful to change either the parasite or the bacterial pathogen, and determine the outcome of anthelminthic on bacterial colonization.

We carefully considered this suggestion, but felt that it would not enhance our manuscript to both broaden the scope by altering either the parasite or bacterial pathogen, while at the same time adding additional mechanistic data specific to the *H. polygyrus-Salmonella* relationship as you suggest above. As we discuss in our manuscript on **page 18 line 335-345**, previous literature on host-bacterial relationships have suggested that the mechanisms governing their interactions vary drastically between different species, and we did not feel it would be appropriate to investigate a different host-bacterial relationship without the same level of detail to which we have investigated the *H. polygyrus-Salmonella* relationship, which would be years of additional experiments.

In summary, the data are convincing and there is no inherent flaw in the work, it, in my view, simply does not go far enough and really needs to be complemented by some analysis of mechanism.

We hope that you will now find that our extensive additions to our manuscript (**new data in Supplemental Figures 1, 2, 7, 8 and 9**) as well as the clarifications and modifications we have made, have enhanced our manuscript to your satisfaction.

Reviewer #2: Main comments are provided in the above sections. Overall, this was a rigorously-conducted study, yielding interesting information of public health relevance, however, further exploration of the requirement for invasive Salmonella in the bacterial persistence phenotype when H.polygyrus is removed after bacterial inoculation would be useful.
Two experiments are needed (as described in previous sections):
- consistently measure fecal egg burdens in untreated vs PP treated mice to validate deworming for Figs 1 and 4
- Use of invA Salmonella for the 'after inoculation' PP-treatment

Many thanks for your positive comments and experimental suggestions. As we described in detail in our response to your comments above, we have clarified our reporting of the efficacy of our deworming treatment in Figures 1A and for data presented in Figure 4, in **new Figure S7**. As suggested, we completed a new set of experiments to determine the requirement of invA expression by *Salmonella* to persist following PP-treatment, as well as an alternative experiment to test whether tissue invasion contributes to *Salmonella* persisting after deworming treatment, and the new data obtained from these experiments are now reported in **new Figures S8 + S9**.

Once again thank you for facilitating this review process. We believe our newly added data and amendments have significantly improved and strengthened our manuscript, which we hope you will now judge to be fully suitable for publication.

Yours sincerely,

Lisa A. Reynolds, Ph.D.
Assistant Professor
Dept. of Biochemistry & Microbiology
University of Victoria
